# Can myopia be prevented?

**Published:** 2019-05-13

**Authors:** Krupa Philip, Xiangui He, Padmaja Sankaridurg

**Affiliations:** 1 Research Scientist: Brien Holden Vision Institute, Sydney, Australia.; 2 Independent researcher in eye disease prevention and treatment.; 3 Head: Global Myopia Centre, Brien Holden Vision Institute, Sydney, Australia.

**Increasing children's time outdoors, and reducing near work, can delay the onset of myopia – which reduces the risk of high myopia and its complications.**

**Figure:**
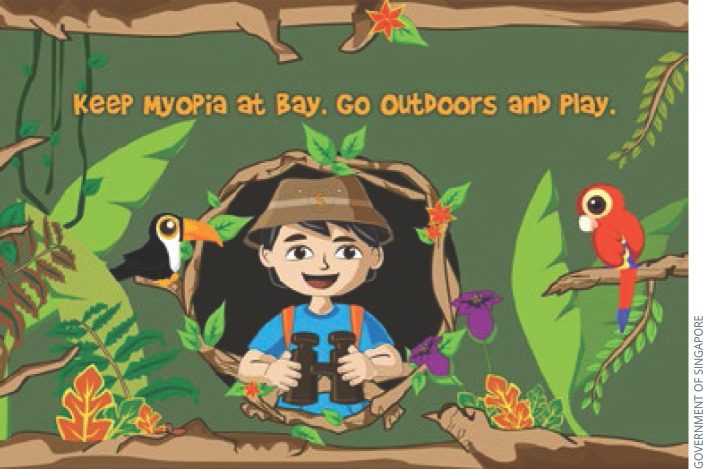
Posters by the government of Singapore encourage children to spend more time outdoors. SINGAPORE

Children who develop myopia at an early age have a greater risk of eventually developing high myopia (≤ −5 dioptres [D] of correction). High myopia increases the risk of retinal detachment, macular degeneration, open-angle glaucoma and cataract. One of the most cost effective ways to reduce this is to prevent or delay the onset of myopia.

The rapid rise in myopia prevalence in recent decades is likely due to environmental factors, such as living spaces (urban versus rural locations),^1^,^2^ indoor versus outdoor activity^3^,^4^ and increased near work.^5^ However, it may be possible to modify these environmental factors in order to reduce or delay the onset of myopia. As early onset of myopia results in high levels of myopia, due to faster ocular growth and greater annual progression during the developmental years,^6^ intervention at an early age also appears to be critical.

## What is the main cause?

Children who perform more near work are more likely to have myopia, and the odds of this increases with additional near work activities each week.^7^ Although the exact mechanism is unclear, researchers suggest that hyperopic defocus from accommodative impairment could be a factor.

Interestingly, studies have also found that more time outdoors delays myopia onset.^8^ Exposure during school recess to outdoor light (sunlight) totalling 11.2 hours per week^9^ or exposure to light in school hallways or under the trees for a longer duration (3.3 hours per day)^10^ has been effective at reducing myopia incidence. Similarly, previous data involving Caucasian and East Asian children growing up in Australia, Singapore and United States found a total of 10–14 hours per week of outdoor time was effective for delaying myopia onset.^4^,^11^ A recent longitudinal study conducted in Australia evaluated children's light exposure using a wearable measuring device; it observed slower eye growth in children exposed to more light each day.^12^ The intensity of artificial indoor light is substantially less than outdoors,^13^ and it also consists of a different spectrum of frequencies. The levels of dopamine, which may play a role in inhibiting eye growth, have also been shown to increase with exposure to light.^14^

Evidence suggests that a multi-pronged approach with reduced near work (especially for young children), living spaces with more natural light as well as promoting time outdoors could be beneficial in delaying or preventing myopia onset. These strategies also have other health benefits, such as reduced risk of obesity and improved physical activity and mental health, so all children will benefit if these are implemented.
